# Developing a tool to assess the skills to perform a health technology assessment

**DOI:** 10.1186/s12874-022-01562-4

**Published:** 2022-03-22

**Authors:** Julia Bidonde, Jose Francisco Meneses-Echavez, Brian Asare, Lumbwe Chola, Mohamed Gad, Lieke Fleur Heupink, Elizabeth Fleur Peacocke, iDSI Ghana HTA collaboration Group, Angela Ackon, Akuba Dolphyne, Francis Ruiz, Ingvil Sæterdal, Anne Lien Espeland, Eia Elena Skjønsberg, Marit Johansen

**Affiliations:** 1 Norwegian Institute of Public Health, P.O. Box: 222 Skøyen, 0213 Oslo, Norway; 2 School of Rehabilitation Sciences, College of Medicine, University of Saskatchewan, Suite 3400, 3rd Floor, 104 Clinic Pl, Saskatoon, SK S7N 2Z4 Canada; 3 Facultad de Cultura Física, Deporte y Recreación, Universidad Santo Tomás, Bogotá, Colombia; 4 Ghana Ministry of Health, Ministries Accra, P.O.Box M 44, Accra, Ghana; 5 London School of Hygiene & Tropical Medicine, Keppel St, London, WC1E 7HT UK

**Keywords:** Health technology assessment, Competence, Capacity building, Tool, Developing countries, Low and middle income countries, Skills, Research

## Abstract

**Background:**

Health technology assessment (HTA) brings together evidence from various disciplines while using explicit methods to assess the value of health technologies. In resource-constrained settings, there is a growing demand to measure and develop specialist skills, including those for HTA, to aid the implementation of Universal Healthcare Coverage. The purpose of this study was twofold: a) to find validated tools for the assessment of the technical capacity to conduct a HTA, and if none were found, to develop a tool, and b) to describe experiences of its pilot.

**Methods:**

First, a mapping review identified tools to assess the skills to conduct a HTA. A medical librarian conducted a comprehensive search in four databases (MEDLINE, Embase, Web of Science, ERIC). Then, incorporating results from the mapping and following an iterative process involving stakeholders and experts, we developed a HTA skills assessment tool. Finally, using an online platform to gather and analyse responses, in collaboration with our institutional partner, we piloted the tool in Ghana, and sought feedback on their experiences.

**Results:**

The database search yielded 3871 records; fifteen those were selected based on a priori criteria. These records were published between 2003 and 2018, but none covered all technical skills to conduct a HTA. In the absence of an instrument meeting our needs, we developed a HTA skill assessment tool containing four sections (general information, core and soft skills, and future needs). The tool was designed to be administered to a broad range of individuals who would potentially contribute to the planning, delivery and evaluation of HTA. The tool was piloted with twenty-three individuals who completed the skills assessment and shared their initial impressions of the tool.

**Conclusions:**

To our knowledge, this is the first comprehensive tool enabling the assessment of technical skills to conduct a HTA. This tool allows teams to understand where their individual strengths and weakness lie. The tool is in the early validation phases and further testing is needed.

**Trial registration:**

Not applicable.

**Supplementary Information:**

The online version contains supplementary material available at 10.1186/s12874-022-01562-4.

## Background

Country efforts to improve Universal Health Coverage are increasingly focused on improving the uptake of health technology assessment (HTA) especially in low- and middle-income countries (LMICs), which are increasingly showing more commitment towards ensuring the efficient and equitable delivery of essential health services to all citizens [1]. The goal of HTA is to provide input to decision-making in policy and practice and ensure value-for-money. Health technologies include medical devices, medicines, vaccines, procedures, health services, tests, programs or systems and public health interventions. Evidence from resource-constrained settings suggests that organizations and institutions may have limited capacity to produce and use evidence in decision-making, though there is a growing demand for the development of specialist skills, such as those required to conduct a HTA [2]. In this study, we used the latest definition of HTA agreed by the International Network of Agencies for Health Technology Assessment [3] (Table 1 presents the operational definitions used by the Team).

**Table 1:** Concepts and definitions

Concept	Definition
Capacity building	The process by which individuals and organizations develop or strengthen abilities related to understanding, providing input to, conducting, or utilizing HTA for health policy and decision making, as well as, developing awareness and support in the environment within which HTA is being used [4].
Critical appraisal	Critical appraisal is the process of carefully and systematically assessing the outcome of scientific research (evidence) to judge its trustworthiness, value and relevance in a particular context. Critical appraisal looks at the way a study is conducted and examines factors such as internal validity, generalizability and relevance [5].
Ethics	Health care is a moral endeavour, and the vast potential of technology poses complex moral challenges. A thorough assessment of technology would include reflection on these moral aspects. Ethics provides such a moral reflection [6].
Evidence-Based Practice (EBP)	EBP is a problem-solving approach to the delivery of health care that integrates the best evidence from studies and patient care data with clinician expertise and patient preferences and values. When delivered in a context of caring and in a supportive organizational culture, the highest quality of care and best patient outcomes can be achieved [7].
Evidence synthesis	Evidence synthesis is a way of combining information from multiple studies that have investigated the same thing, to come to an overall understanding of what they found. This helps determine how effective a certain treatment or drug is, or how people have experienced a particular health condition or treatment [8].
Health Technology Assessment	“HTA is a multidisciplinary process that uses explicit methods to determine the value of a health technology* at different points in its lifecycle. The purpose is to inform decision-making in order to promote an equitable, efficient, and high-quality health system.” [3] *A health technology is an intervention developed to prevent, diagnose or treat medical conditions; promote health; provide rehabilitation; or organize healthcare delivery. The intervention can be a test, device, medicine, vaccine, procedure, program, or system.
Patient and Public Involvement (PPI) in HTA	PPI is defined here as the incorporation of the views and perspectives of those who use or are affected by technologies into the assessment of these technologies. In HTA, PPI can take many forms, including inviting patients to join expert panels, to provide evidence mediated by an interviewer or survey, or to provide written submissions about the technology or condition being considered [9, 10]
Stakeholder	Individual or group that has an interest in any decision or activity of an organization.” Stakeholders may include suppliers, internal staff, members, customers (including shareholders, investors, and consumers), regulators, and local and regional communities. Additionally, stakeholders may include purchasers, clients, owners, and non-governmental organizations [11].

HTA is a policy tool, as it generates evidence to inform policy decisions and practice. The World Health Organization (WHO) proposed that HTA needs to be seen as a set of skills, and needs to be developed in countries in a way that is, from the beginning, linked to decision-making [12]. This aligns with a summary report of published literature on capacity building for skills related to HTA in the Republic of Ghana (hereafter Ghana), which found that there is a need to develop HTA expertise within Ghana to conduct locally relevant HTAs [13–15]. Key success factors for HTA implementation include building human resources and (financial) capacities, establishing a transparent decision-making process, and implementing robust HTA methodology [16]. A system’s capacity for HTA relies on performance (e.g. equipment) and personal capacity (e.g. technical skills), infrastructure (e.g. data availability and management), resources expertise (e.g. staff) in related disciplines and collaborations. Further, it requires structural capacity (e.g. planning systems, inter sectoral forums) to ensure research quality and provide protection from vested interests, and research grants, all of which are limited in LMICs [17].

The literature, however, points at important challenges faced by LMICs in institutionalizing HTA. In a study of selected countries in Asia and Africa, Kim et al. [18] suggests that there are three main challenges faced by countries namely lack of technical capacity, funding, and stakeholder engagement. Uzochukwu and colleagues [19] call for interventions to improve capacity in HTA as deficiencies in knowledge and skills in HTA exist. They noted a lack of awareness for HTA and human resource capacity for conducting and interpreting HTAs. Rosselli’s study [20] emphasized the increasingly important role of HTA in Latin American countries. Authors reported insufficient human resource and public investment for HTA implementation in Latin America [21].

The catalyst for this project was a collaboration between Ghana’s Ministry of Health and the Norwegian Institute of Public Health (NIPH) as partners of the International Decision Support Initiative (iDSI), supporting institutionalization of HTA in Ghana (from now on referred to as the Team). Ghana has been a leading African nation for Universal Health Coverage, and was one of the first nations on the African continent to enact legislation that established a social health insurance model. Ghana’s National Health Insurance Scheme (NHIS) Act (Act 650) was passed in 2003 administered by the National Health Insurance Authority. The Ghanaian Minister of Health inaugurated the HTA Steering Committee, HTA Technical Working Group (TWG) and HTA Secretariat, to oversee the development of HTA under the auspices of the Ghana Ministry of Health in October 2019 [22]. Since 2009 the NHIS has often been in financing deficit; Hollingworth et al. argued that HTA could play an important role in Ghana to support evidence-based priority setting [23].

**Fig. 1:**
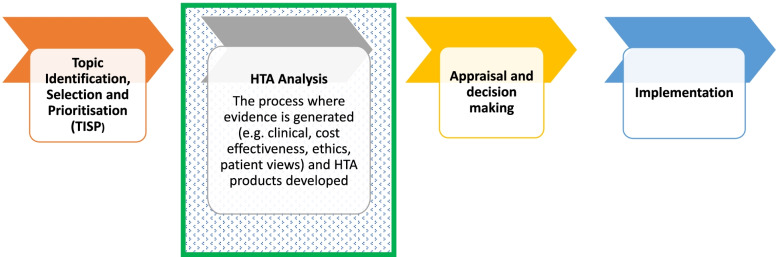
Norwegian Institute of Public Health HTA areas of engagement with LMICs [28]

HTA is used as a primary decision support aid in many countries as it enables the most systematic, transparent, and evidence-based approach towards comparing alternative interventions based up on pre-defined criteria of interest to decision-makers [24, 25]. However, the use of HTA in emerging economies may focus more on the inclusion of health economic evaluations [26, 27], and be less on the means to identify data related to the effectiveness of the intervention, patient and public engagement, ethics or organizational challenges. The NIPH’s approach to HTA processes includes a wide set of necessary technical skills, including a combination of information retrieval, evidence synthesis, interpreting clinical research, health economic evaluation, ethics, patient engagement, appraisal and decision making (see ‘HTA Analysis’ in Fig. 1) [28].

To support HTA in Ghana, the Team sought to understand the in-country individual technical capacity, analyzing existing capacities and generating an understanding of resources and needs. The team recognized that Ghana had knowledge and skills to offer, and any future capacity building activities had to capture, capitalize and build on and those pre-existing skills. Information would serve as input for formulating a capacity development response to strengthen and optimize existing capacities. It would also set the baseline for continuous monitoring and evaluation of progress against relevant indicators and help create a solid foundation for long-term planning, implementation and sustainable results.

The team was not aware of any existing validated tools for this purpose, and tools that we were aware of, were for example the “WHO Global Survey on Health Technology Assessment by National Authorities” [29], which focused on system level or institutional capacities for HTA, rather than the individual which was necessary for NIPH’s long-term collaborations. Other tools the team was aware of focused only on health economics [30] or patient and public engagement [31]. In addition, a summary report of published literature related to capacity building and HTA in Ghana had identified strength in primary research, information retrieval, and analysis [13], but that no specific assessment HTA assessment had previously been completed in Ghana. Therefore, the Team initiated the project with a mapping review to develop an individual skills assessment tool that could be used in various settings. In this article, we present details of the methods used to develop the HTA skills assessment tool, as well as some lessons learnt from piloting out the tool on the Ghanaian HTA TWG.

## Methods

### Aim, design and setting of the study

The aims of the project were:

a) to identify existing tools intended to assess the technical capacity needed for the conduct of HTA (i.e., information retrieval, evidence synthesis, interpreting clinical research, health economic evaluation, ethics, patient engagement) and in the event of not finding one tool, to develop a tool to assess the capacity to conduct HTA, and.

b) to describe experiences of piloting the tool with the Ghanaian HTA TWG.

**Fig. 2:**
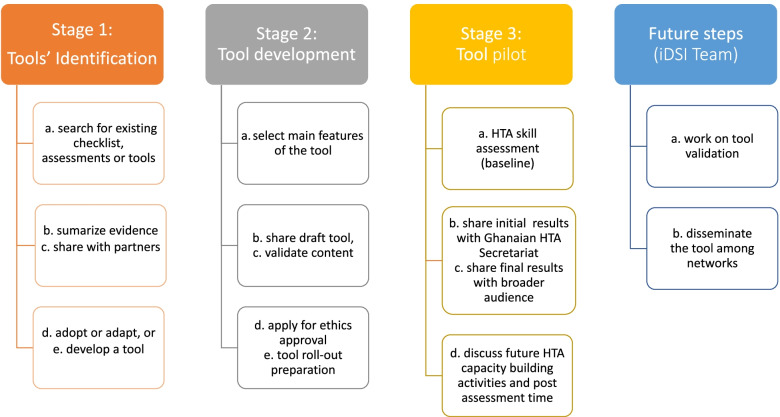
Design of the study

The study was developed through an iterative, collaborative and consensus-based process designed to ensure relevance and acceptability of the final product to a broad audience. We conducted the project in three stages as Fig. 2 shows:

### Stage 1 – tools’ identification

First, we conducted a mapping of the existing literature and searched for checklists, evaluations, assessments or tools (hereafter tools) assessing the capacity of undertaking HTAs in LMICs. We followed the methodological guidance for scoping reviews from Arksey and O’Malley [32] and Levac and colleagues [33], and the Preferred Reporting Items for Scoping Reviews (PRISMA-ScP) statements [34]. The protocol for this mapping review was not registered.

### Information sources and literature search

A health information specialist conducted a search in MEDLINE, Embase, Web of Science, and ERIC databases; the search strategy was peer reviewed in line with the Peer Review of Electronic Search Strategies (PRESS) framework [35]. Search terms used related to ‘capacity building,’ ‘health technology assessment’, and ‘low- and middle income countries’. The search was restricted to years 2000 to August 2019 and used the LMICs filter. Additionally, we searched grey literature via Google Scholar, and hand searched selected bibliographies. Additional file 1 presents the final search strategy for MEDLINE database and grey literature sources.

### Study selection process

After de-duplication a single researcher (JB/JFME) screened titles and abstracts independently and together agreed on full text inclusions. Discrepancies were resolved through discussion. The screening criteria were established a priori, and was calibrated between reviewers. Selection criteria were to include:

- records that presented a tool. A tool was defined as an instrument or set of criteria used to evaluate the ability, knowledge and/or capacity (hereafter skills) to perform a HTAs either in full or any of its parts (i.e., clinical effectiveness synthesis, economic models) [36];
- records (e.g., organizational reports or journal articles) that described a tool used to assess knowledge, skills or capacity in the fields of evidence synthesis, evidence-based practice, HTA, and health economic evaluations.
- Inclusion was limited to documents that the team was able to translate (i.e., English, Spanish, French, Norwegian or Swedish).

Documents were excluded that focused on description of skills or related concepts. See Table 1 operational definitions used by the Team.

### Data items and data extraction process

The same two researchers mentioned above conducted a content analysis [37] mapping out relevant tools. Articles were classified by established categories following inclusion criteria (Table 2). Researchers independently abstracted data classified the included full texts, all discrepancies were resolved by a third team member (EP).

**Table 2:** Categories for record classification

Category	Description
Methods	The record focused on research methods for the conduct of an HTA (e.g., literature searches or critical appraisal of primary studies). For example, a record aiming to evaluate the capacity for conducting systematic reviews in a LMIC setting.
Capacity building or assessment	The record described either an intervention, strategy, educational initiative, or framework related to capacity assessment/building of health economics, clinical effectiveness, patient involvement or patient and public involvement in the context of an HTA. For example, a step-by-step guide to conducting a capacity assessment, or lessons learnt from programs.
Evidence-based practice	The record focused area was in EBP and proposed methods to evaluate skills in this area. For example, presents tools to assess EBP learning and teaching.
Ethics	The record described ethical considerations in a HTA process or skills needed in this field. For instance, detailed guidance on how and when to incorporate ethical considerations within an HTA.

Partners in the iDSI network (i.e., from Norway, United Kingdom and Ghana) attended a three-day meeting in Oslo (October 2019) and discussed preliminary results derived from the mapping review, and that a tool had not been identified that focused on the assessment of all skills required to perform a HTA. Thus, the discussions led the partners to agree, by informal consensus, to develop a tool to measure the needed skills to conduct a HTA. This study responds to this gap by developing a generic evaluation tool for use in a wide range of organizations.

### Stage 2: development of a tool

The tool was developed through an iterative, collaborative process informed by a review of published and grey literature and with the input of researchers and HTA experts. First, we defined that the purpose of the tool was to evaluate technical capacity (e.g., skills in information retrieval, evidence synthesis, interpreting clinical research, health economic evaluation, ethics, patient engagement) to conduct HTAs. The next step in the tool development process involved mapping the core principles to outcome measures that would serve as the starting point for the creation of the tool. The partners agreed the tool would have four categories: General information, Core skills, Soft skills, and Future needs. *General information* included demographic questions related to professional experience and experience with HTA. *Core and soft skills* came from the identification of specific skills, and the proposal that the tool should incorporate basic skills (e.g., ability to prepare reports and documents, communication, professionalism) or cross-functional specific skills (e.g., ability to critically appraise literature, understanding of the HTA process); respectively, we referred to these as “soft” and “core” skills [38]. We defined *soft skills* as personal competences not directly linked to a specific task. For example, social aptitudes, language and communication, friendliness, and ability to work in a team, political awareness, and leadership. *Core skills,* on the other hand, were defined as the specific abilities to perform a HTA task or activities related to HTA [38, 39], such as review of clinical effectiveness, literature searches, or economic evaluations. Soft and core skills sections of the tool were thought to be supplemented with current experiences of the respondents and a future needs section. *Future needs* section is designed to help understand areas that respondents are interested in pursuing For each of these categories, a set of measurable outcomes, also informed by the literature was generated.

**Fig. 3:**
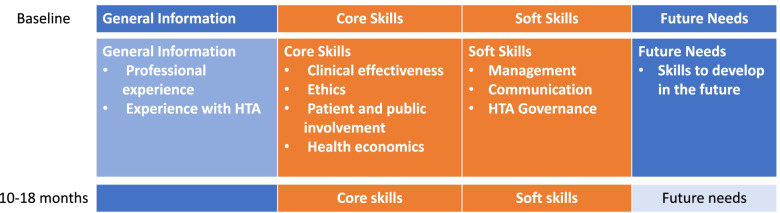
HTA skills assessment tool’s initial configuration

Construction of the draft tool followed this iterative and collaborative process and was informed by a bank of sample questions compiled from evaluation resources identified in Stage 1. Relevant questions were used and/or adapted as necessary. Figure 3 shows the tool’s initial configuration including data gathering times (i.e., baseline, follow up assessment), areas (i.e., general information, core and soft skills, and future needs) and tentative time to complete it (i.e., 10 to 30 min). While the pre-assessment (baseline) would include the four components, the follow up-assessment (10–18 months) would include core and soft skills leaving the future needs section and the general information sections as optional.

The planned target audience for the tool was anyone involved in the development and production of a HTA to inform policy decisions across various sectors (government department, agencies, academia).

### Validity of the tool

An important aspect that arises when developing an instrument is its quality. To ensure content validity (i.e., the appropriateness of the subject matter), as stated previously, we used an iterative process to determine the final list of items in the newly developed tool and its scoring. We sought expert opinion from partners at NIPH and iDSI (i.e., Ghana, Norway, and UK) from individuals with extensive experience with HTA and regarded as experts in their fields. These individuals were asked to provide feedback on the structure, layout, comprehensibility, ease of use and utility of the tool to relevant end users. An additional objective of the expert opinion consultation was to balance the number of items in the assessment tool, maintain practicality of administering and retaining the tool’s discriminative ability. The tool was further revised and presented in Accra, Ghana (December 2019), where it was agreed the final tool would be piloted with the Ghanaian TWG. The tool was set up as an online questionnaire using the electronic platform Questback (Questback GmbH Cologne, Germany) [40].

#### Scoring and analysis

The four sections of the tool have been described previously (see Fig. 3); the scoring for each is as follow:

The *General Information* section contains a total of 22 statements (see Additional file 2). The *Core Skills* section contains four sub-sections which consist of statements that investigate the needed skills in areas commonly included in a (full) HTA. The first sub-section, *clinical effectiveness,* is further sub-divided into skills for *planning* a HTA (9 statements) and *doing* a HTA (45 statements). The ethics and patient and public involvement sub-sections contain three and four statements each. In the fourth, and last sub-section informants are asked about their skills in health economics (11 statements); these statements were adapted (with permission) from the iDSI economic tool [30]. Each statement seeks informants to reflect and respond with what the individual knows about topic and what level of skills she/he has in the area. The *Soft skills* section contains 12 statements including the areas of project coordination and management, leadership, resourcefulness, dialogue and negotiations, networking, teamwork and adaptability/flexibility. The last section of the tool explores informants’ *Future needs;* it asks informants to rank the skills presented (8 statements) in order of importance. The tool concludes with an open question (“other needs”) about any future needs that was not covered in the options presented.

The tool allows for the provision of an individual capacity profile; however, at the time of developing we believed it was more useful to provide an overview of the current experience and existing capacities among informants (as a group). The results can be analysed using descriptive statistics. Frequencies and percentages can be used to explain the demographic characteristics of respondents (and means, median and quartiles, and rankings to analyse respondents’ level of skill). All text-data should be transformed to integers (number data type). For instance, ‘yes’ or ‘no’ answers were transformed to ‘1’ and ‘0’ respectively. Results can be presented the according to the tool four areas (i.e., general information, core and soft skills and future needs), and within the results one can combine or aggregate multiple statements to give comprehensive overviews. The *Future needs* statements can also be aggregated and presented in the 8 categories (see Additional file 2).

### Stage 3: piloting of the tool

After receiving ethical approval, the Ghana HTA Secretariat initiated contact with members of the TWG requesting to assess their technical HTA skills, which was followed up by an email from the Norwegian team. In the email invitation, we described the aim of assessment, approximate time to completion, and instructions to fill in the questionnaire. Given the size of the group and the risk of informants’ identification, the results of the tool pilot are not part of this report and were exclusively shared within the Norwegian team and Ghanaian partners.

## Results

### Stage 1: identification of existing tools

The database search yielded 3871 records, and additional searches added 32 records. After removal of duplicates, we screened 2676 records at title and abstract. Fifty-seven of those were promoted to full text screening; finally, 15 records were included (see Additional File 3 PRISMA flow-chart). The 15 documents retrieved fit the inclusion criteria, however, they were peripherally related, and none of the tools dealt specifically with HTA skills assessment. The tools were published between 2003 and 2018; each single tool covered one category (e.g., capacity building/assessment or evidence-based practice), except three documents [41–43]. Nine tools focused on evidence-based practice and methods [41–49], whereas other nine tools covered topics related to capacity building assessment [14, 15, 29, 41–43, 50–52]. We did not identify any tools to assess skills in ethical, organizational or patient involvement areas. Five tools (33%) used a cross-sectional design [29, 43, 46, 49, 50]. The tools were directed to various target groups, mostly researchers. Additional File 4 presents the characteristics of the 15 included documents.

These 15 tools evaluated some of the skills of interest and provided items that contributed to the development of the final tool. For example, The United Nations Development Programme (UNDP) [51] presented the Capacity Assessment Methodology, which provides a detailed step-by-step guide to conducting a capacity assessment using the UNDP Capacity Assessment Framework, a three-step process and supporting tools. This tool pointed out the five steps of the UNDP capacity development process some of which considered (I.e., 1. engage stakeholders on capacity development; 2. assess capacity assets and needs; 3. formulate a capacity development response; 4. implement a capacity development response; 5. evaluate capacity development). Similarly, Watson-Grant et al. [52], as part of the MEASURE evaluation developed the Evaluation Capacity Strengthening Framework and facilitated a guidance document for assessing and planning evaluation capacity strengthening. Regarding the EBP category, Oliver et al. [46] conducted a rapid appraisal of tools to evaluate the capacity for conducting systematic reviews in LMICs. In the Ghanaian context, Bates et al. [44] developed a tool for assessing research capacity through the analysis of published models and effective capacity-building principles alongside active involvement of key stakeholders at the Komfo Anokye Teaching Hospital in Kumasi, Ghana.

### Stage 2: developed tool for assessing HTA skills

Conscious of constrained resources in LMICs settings, we designed the tool to be administered to a broad range of individuals (including those who are experienced at economic or clinical reviews, searching for literature, and managing ethical and patient engagement) who would potentially contribute to one or more activities related to the planning, delivery and evaluation of HTA. This tool is freely available on the NIPH website [40] and presented in Additional file 2. As described in the scoring section, the final tool has the following four sections (Fig. 3):

The *General Information* section gathered data on informant’s experiences with research, government positions, or HTA. This section asked general demographic information (i.e., age, gender) and specific questions about years of professional experience, collaborations, area of expertise, use of research or HTA, experience with synthesis, health economics, and type of role (i.e., project leader, collaborator, stakeholder). The *Core Skills* section is the longest part in the tool. This section includes skills needed in four areas commonly included in a (full) HTA, such as clinical knowledge, health economics, ethics and patient involvement. The *soft skills* section contains statements regarding skills in the areas of project coordination and management, leadership, resourcefulness, dialogue and negotiations, networking, teamwork and adaptability/flexibility. The last section of the tool explores informants’ *future needs* (skills to develop in the future) with regards to their core skills.

### Stage 3: pilot of the tool

Twenty-three individuals who received the invitation to participate, completed the skills assessment. Bounded by our ethical commitment, the Norwegian team and Ghana partners only have access to the analysis and results of the assessment. We shared preliminary results with the Ghana Secretariat in first instance, to discuss the relevance of the information, then we presented the final results to a broader audience (i.e., TWG, WHO Ghana, MoH). Our observations throughout the development and use of the tool speak of the usability of the tool, the user’s values, and context of use (e.g. time, place). These observations enabled us to provide the reader with initial impressions of the users and team experiences.

- Participants indicated that the tool was easy to use, had a clear purpose, helpful headings/instructions, and was easy to understand and useful to the organization.
- With the emphasis on comprehensiveness, we heard the tool is long; we understood from experts and informants alike that a shorter version would be preferable. However, all informants agreed that the assessment although it was lengthy, it was worth the time.
- Although the catalyst for this project was the iDSI collaboration, the tool was not developed exclusively for LMICs and it may be valuable for other settings; we acknowledge, however, it may be more suitable in contexts were multiple (HTA) functions are performed by the same individual.
- The tool is set up to be completed in one time (the current online platform does not have a ‘save’ feature), this brought two main remarks: a. the individual completing the assessment is focused and data provided could be trusted and b. fatigue can be a factor affecting completion or accuracy of final sections of the tool.
- We gathered during the presentation of the draft tool (Accra 2019), that there was some initial wariness to “*being assessed*”; however, oral feedback after the tool pilot (June 2021) validated the fact that the skill assessment was a “useful and humble” exercise. It provided confirmation to us all about the many skills the TWG (individual) group possess as well as areas to focus on in the future.
- Setting up the tool online was an easy and practical way to gather baseline data; it allowed direct access to the individuals during the 2020–21 pandemic. Providing the informant had internet access, the skills assessment could be completed from their preferred location and device.
- We heard the tool itself, through its structured overview of core aspects of HTA, brought informants’ capacity self - awareness (i.e. an overview on his/her own skills). Initial reactions to baseline results need to be managed in a positive and meaningful environment.
- Initial results from the pilot appear to have a strong practical application. Results have been considered in planning learning processes intended to enhance Ghana’s HTA skills.

## Discussion

As efforts to achieve Universal Health Coverage have grown in LMICs, particularly with some countries introducing social insurance models, increasing attention has also been paid to implement HTA to ensure the efficient and equitable delivery of essential health services to all. However, the ability of countries to manage and implement HTA is dependent on processes being institutionalized, building knowledge and skills and improving organisational capacities across institutions and partners. Assessment of HTA skills is an important step for countries formalising and understanding HTA capacities. Although we do not make any claims to exhaustiveness, this is, to the best of our knowledge, the first tool to assess technical HTA skills capacity. However, there are a few weaknesses of the tool development process that warrant further attention. First, we sought to balance rigour and applicability, but we might have focused on our Ghanaian’s partners needs throughout the tool development process. This may have yielded a less robust tool with respect to its validity and reliability (i.e., psychometric properties). Furthermore, the tool has not yet been applied outside a small group in Ghana, and further guidance may be needed in order to expand and adapt the tool to other jurisdictions. By focusing on this small group, some specificity may have been lost that may be important to other organizations or individuals and that would necessitate further adaptation. These weaknesses may be counterbalanced by our extensive review of the literature and by the international expertise brought by the iDSI partners. We are aware the tool is an early stage of development, and we look forward to the its path of continuous improvement and refinement.

It is known that HTA is multidisciplinary, and those involved in HTA need strong research (methods, health economics, epidemiological, search, ethics, etc.) skills [53] . Although the catalyst for this project was our collaboration with Ghana, the tool described here was not developed exclusively for LMICs and it may be valuable for other settings; we acknowledge, however, it may be more suitable in contexts were multiple (HTA) functions are performed by the same individual. Potential users of this tool include evaluators, researchers, policy and program decision-makers, as well as any person, group, or organization wanting to know their individuals’ skills to conduct a HTA. One of the important contributions of this tool is that when it is implemented, the tool creates an opportunity to share experiences and knowledge with other institutions who are interested in similar projects.

An assessment plays a critical role in a HTA organization helping improve performance, increasing accountability for results, and promoting organizational learning. Assessments’ results should influence policy and operational decisions [54]. It not only leads to a clearer understanding of the financial return on educational programs [55], but also allows its developers to design programs that permit informants to achieve their highest level of learning [56]. To define what and whose capacities are to be developed through and intervention or project, it is necessary to have a baseline assessment on existing capacities and gaps. This provides an opportunity for strategic (and targeted) planning and management of capacity development opportunities. In that regard, our tool has important potential impacts especially for those involved in policy decision maker in resource constrained settings: first the tool gives a baseline assessment of individual (or group) skills allowing personalized target capacity development, second, it provides an opportunity to have a collaboration in defining future capacity development strategies and scope. Using the proposed tool should not require a lot of planning by the group interested in measuring skills to conduct a HTA.

There has been steady progress in LMICs health research capacity, nonetheless major barriers to research in these settings persist [45]; chronic underinvestment in universities and research institutions, low wages and poor career prospects for researchers push many researchers opting to work abroad or to devote more time to other activities such as teaching and consultancy [57]. Owing to the multidisciplinary nature of HTA, researchers should have a broad training and understanding of several topics and methods. Countries’ strengthening of evidence to decision processes, including HTA, are contingent on locally led evidence synthesis, health economics, and evidence appraisal; the HTA field could provide career opportunities for locally trained researchers. The opportunity that HTA skills provide for evidence-informed allocations of scarce health resources must also consider that the changes at individual level either in capabilities, skills, and knowledge per se cannot do much if not seen without the institution and organization, structure, and leadership – and with alignments in governance and policy to support [58]. The Norwegian team has been contacted by individuals from other LMICs regarding the availability of tool, which has confirmed the need for such instrument and added value to our work; engagement of other countries will provide further opportunities for validation.

### Tool future plans

The results of the assessment are only as valid as the tool itself; thus, further consideration should be given to the validity and reliability of the assessment tool itself. The team has planned to conduct a factor analysis to explore the underlying factor structure of the tool, that is, whether the tool is measuring the proposed topics as intended, and whether some items can be combined. Factor analysis will also help to uncover the minimum number of items (statements) required per factor; results may help to shrink the tool to a smaller and more manageable version. Following the factor analysis, the end results may be a shorter version of the tool, but results may also suggest a reorganization of questions. Concurrent validity could be tested by collecting semi-structured qualitative data and analysing the results to assess consistency of individual responses to the tool. At this stage, the team plan to spend some time into understanding the results of the factor analysis and qualitative data deciding on items to keep the way they are or modify.

### Limitations

There are some limitations to the methodological approach that we undertook, largely related to the relatively short timeframe to conduct a mapping review, develop and revise the tool, and pilot it. We acknowledge that we approached the literature with a predefined idea of the HTA process, and this could have influenced the selection of studies included in the mapping review and the richness of our content analysis. In addition, we note that given that the context of this project was related to using HTA for Universal Health Coverage in Ghana, we were guided by the HTA skills more closely related to organisational interventions, differing from many high-income settings where many HTAs are examining high-tech interventions. An important limitation is the proper validation of the tool, however initial feedback on content, domains and format has been positive. While the tool appears to have face validity, implications for use of the tool in practice are cautioned until further evidence is available. In order to get robust results, we recognize further work on the validity and reliability of the instrument is warranted. An important limitation of the pilot is that informants were a selected group. For this reason, one must assume that this group exhibited different motivation levels and perhaps are different from Ghanaians involved in HTA.

## Conclusions

An extensive literature review found no previous tools for evaluating skills to conduct a HTA; this project developed one. The instrument itself facilitates an opportunity for HTA teams (or individuals) to understand were their strength and weakness lie and to ensure any future capacity building activities are framed and planned in a way that is most relevant to their members. Initial pilot of the tool has begun and further testing is needed. However, further steps for gathering evidence in its validity and reliability are in the planning stages. We welcome feedback from those who want to use the toll, and their results to inform further refinement of the tool.

## Supplementary Information

**Additional file 1.**

**Additional file 2.**

**Additional file 3.**

**Additional file 4.**

## Data Availability

The *Instrument for the assessment of skills to conduct a Health Technology Assessment* is publicly available for use on the Norwegian Institute of Public Health’s website (https://www.fhi.no/globalassets/dokumenterfiler/global-helse/evidence/bidonde-j-et-al-2021-instrument-for-the-assessment-of-skills-to-conduct-hta.pdf).

## Abbreviations

CB: Capacity building
EBP: Evidence based practice
HTA: Health technology assessment
iDSI: International Decision Support Initiative
LMICs: Low and middle income country
NIPH: Norwegian Institute of Public Health
PRESS: Peer Review of Electronic Search Strategies
PRISMA-ScP: Preferred Reporting Items for Scoping Reviews
TWG: Technical Working Group
