# Supplementary material for: Developing a tool to assess the skills to perform a health technology assessment
Source: BMC Med Res Methodol. 2022 Mar 22;22:78. doi: 10.1186/s12874-022-01562-4 (PMC8939100; doi:10.1186/s12874-022-01562-4)
Supplement: Supplementary file 1 — Additional file 1. [file 12874_2022_1562_MOESM1_ESM.docx]

**Additional File 1. Search Strategy**

| **Database** | **Date** | **Hits** |
| --- | --- | --- |
| MEDLINE and Epub Ahead of Print, In-Process & Other Non-Indexed Citations and Daily 1946 to August 29, 2019, Ovid | 30.08.19 | 2960 |
| Embase 1974 to 2019 Week 34, Ovid | 30.08.19 | 1031 |
| Web of Science Core Collection, 1987-2019, Clarivate Analytics | 30.08.19 | 263 |
| ERIC, ProQuest | 30.08.19 | 155 |

**MEDLINE Search strategies (searched 30.08.19): MEDLINE and Epub Ahead of Print, In-Process & Other Non-Indexed Citations and Daily 1946 to August 29, 2019, Ovid**

| **#** | **Searches** | **Results** |
| --- | --- | --- |
| 1 | Capacity Building/ | 6575 |
| 2 | exp Education/ | 2134941 |
| 3 | Professional Competence/ | 55107 |
| 4 | Knowledge/ | 46839 |
| 5 | or/1-4 **[CAPACITY BUILDING]** | 2185136 |
| 6 | Evidence-Based Practice/ | 69950 |
| 7 | Evidence-Based Dentistry/ | 2464 |
| 8 | Evidence-Based Medicine/ | 177444 |
| 9 | Evidence-Based Emergency Medicine/ | 767 |
| 10 | Evidence-Based Nursing/ | 7593 |
| 11 | "Review Literature as Topic"/ | 46873 |
| 12 | Systematic Reviews as Topic/ | 2472 |
| 13 | Meta-Analysis as Topic/ | 45048 |
| 14 | Epidemiologic Research Design/ | 197157 |
| 15 | Controlled Clinical Trials as Topic/ | 14466 |
| 16 | Randomized Controlled Trials as Topic/ | 227842 |
| 17 | Technology Assessment, Biomedical/ | 23169 |
| 18 | "Costs and Cost Analysis"/ | 99352 |
| 19 | Cost-Benefit Analysis/ | 159476 |
| 20 | or/6-19 **[HTA]** | 1011312 |
| 21 | 5 and 20 | 95219 |
| 22 | ((capacity or competenc* or knowledge or skill? or qualification? or education or training) adj6 (evidence based or systematic review* or scoping review* or mapping review* or evidence synthesis or metaanalys* or meta analys* or technology assessment* or health economics or cost analysis or cost benefit analysis or economic evaluation or cost effectiveness)).ti,ab,kf. | 23979 |
| 23 | or/21-22 **[CAPACITY BUILDING + HTA]** | 113970 |
| 24 | Developing Countries.sh,kf. | 83947 |
| 25 | (Africa or Asia or Caribbean or West Indies or South America or Latin America or Central America).hw,kf,ti,ab,cp. | 586833 |
| 26 | (Afghanistan or Albania or Algeria or Angola or Antigua or Barbuda or Argentina or Armenia or Armenian or Aruba or Azerbaijan or Bahrain or Bangladesh or Barbados or Benin or Byelarus or Byelorussian or Belarus or Belorussian or Belorussia or Belize or Bhutan or Bolivia or Bosnia or Herzegovina or Hercegovina or Botswana or Brasil or Brazil or Bulgaria or Burkina Faso or Burkina Fasso or Upper Volta or Burundi or Urundi or Cambodia or Khmer Republic or Kampuchea or Cameroon or Cameroons or Cameron or Camerons or Cape Verde or Central African Republic or Chad or Chile or China or Colombia or Comoros or Comoro Islands or Comores or Mayotte or Congo or Zaire or Costa Rica or Cote d'Ivoire or Ivory Coast or Croatia or Cuba or Cyprus or Czechoslovakia or Czech Republic or Slovakia or Slovak Republic or Djibouti or French Somaliland or Dominica or Dominican Republic or East Timor or East Timur or Timor Leste or Ecuador or Egypt or United Arab Republic or El Salvador or Eritrea or Estonia or Ethiopia or Fiji or Gabon or Gabonese Republic or Gambia or Gaza or Georgia Republic or Georgian Republic or Ghana or Gold Coast or Greece or Grenada or Guatemala or Guinea or Guam or Guiana or Guyana or Haiti or Honduras or Hungary or India or Maldives or Indonesia or Iran or Iraq or Isle of Man or Jamaica or Jordan or Kazakhstan or Kazakh or Kenya or Kiribati or Korea or Kosovo or Kyrgyzstan or Kirghizia or Kyrgyz Republic or Kirghiz or Kirgizstan or Lao PDR or Laos or Latvia or Lebanon or Lesotho or Basutoland or Liberia or Libya or Lithuania or Macedonia or Madagascar or Malagasy Republic or Malaysia or Malaya or Malay or Sabah or Sarawak or Malawi or Nyasaland or Mali or Malta or Marshall Islands or Mauritania or Mauritius or Agalega Islands or Mexico or Micronesia or Middle East or Moldova or Moldovia or Moldovian or Mongolia or Montenegro or Morocco or Ifni or Mozambique or Myanmar or Myanma or Burma or Namibia or Nepal or Netherlands Antilles or New Caledonia or Nicaragua or Niger or Nigeria or Northern Mariana Islands or Oman or Muscat or Pakistan or Palau or Palestine or Panama or Paraguay or Peru or Philippines or Philipines or Phillipines or Phillippines or Poland or Portugal or Puerto Rico or Romania or Rumania or Roumania or Russia or Russian or Rwanda or Ruanda or Saint Kitts or St Kitts or Nevis or Saint Lucia or St Lucia or Saint Vincent or St Vincent or Grenadines or Samoa or Samoan Islands or Navigator Island or Navigator Islands or Sao Tome or Saudi Arabia or Senegal or Serbia or Montenegro or Seychelles or Sierra Leone or Slovenia or Sri Lanka or Ceylon or Solomon Islands or Somalia or South Africa or Sudan or Suriname or Surinam or Swaziland or Syria or Tajikistan or Tadzhikistan or Tadjikistan or Tadzhik or Tanzania or Thailand or Togo or Togolese Republic or Tonga or Trinidad or Tobago or Tunisia or Turkey or Turkmenistan or Turkmen or Uganda or Ukraine or Uruguay or USSR or Soviet Union or Union of Soviet Socialist Republics or Uzbekistan or Uzbek or Vanuatu or New Hebrides or Venezuela or Vietnam or Viet Nam or West Bank or Yemen or Yugoslavia or Zambia or Zimbabwe or Rhodesia).hw,kf,ti,ab,cp. | 7417977 |
| 27 | ((developing or less* developed or under developed or underdeveloped or middle income or low* income or underserved or under served or deprived or poor*) adj (countr* or nation? or population? or world)).ti,ab. | 209736 |
| 28 | ((developing or less* developed or under developed or underdeveloped or middle income or low* income) adj (economy or economies)).ti,ab. | 1149 |
| 29 | (low* adj (gdp or gnp or gross domestic or gross national)).ti,ab. | 576 |
| 30 | (low adj3 middle adj3 countr*).ti,ab. | 29632 |
| 31 | (lmic or lmics or third world or lami countr*).ti,ab. | 14676 |
| 32 | transitional countr*.ti,ab. | 380 |
| 33 | or/24-32 **[LMIC]** | 7757572 |
| 34 | 23 and 33 **[CAPACITY BUILDING + HTA + LMIC]** | 14640 |
| 35 | Decision Making/ | 303150 |
| 36 | Health Policy/ | 214722 |
| 37 | Biomedical Research/ | 244573 |
| 38 | Health Services Research/ | 68492 |
| 39 | or/35-38 | 802814 |
| 40 | 5 and 39 | 130084 |
| 41 | ((capacity or competenc* or knowledge or skill? or qualification? or education or training) adj6 (research evidence or scientific evidence or medical research or health research or health services research or health systems or health policy or health care policy or policy making or decision making)).ti,ab,kf. | 22128 |
| 42 | 40 or 41 | 148258 |
| 43 | Ghana/ or ghana*.ti,ab,kf. | 24060 |
| 44 | 42 and 43 **[CAPACITY BUILDING + RESEARCH / POLICY + GHANA]** | 252 |
| 45 | limit 34 to yr="2005 -Current" | 11676 |
| 46 | limit 44 to yr="2005 -Current" | 225 |
| 47 | 45 or 46 | 11878 |
| 48 | 47 use ppez **[MEDLINE RECORDS]** | 2960 |
| 49 | capacity building/ | 6575 |
| 50 | exp evidence based practice/ | 1278806 |
| 51 | biomedical technology assessment/ | 23390 |
| 52 | health economics/ | 32141 |
| 53 | exp economic evaluation/ | 371414 |
| 54 | or/50-53 | 1649564 |
| 55 | 49 and 54 | 776 |
| 56 | ((capacity or competenc* or knowledge or skill? or qualification? or education or training) adj6 (evidence based or systematic review* or scoping review* or mapping review* or evidence synthesis or metaanalys* or meta analys* or technology assessment* or health economics or cost analysis or cost benefit analysis or economic evaluation or cost effectiveness)).ti,ab,kw. | 24544 |
| 57 | 55 or 56 | 25190 |
| 58 | Developing Country.sh. | 91517 |
| 59 | (Africa or Asia or Caribbean or West Indies or South America or Latin America or Central America).hw,ti,ab,cp. | 573837 |
| 60 | (Afghanistan or Albania or Algeria or Angola or Antigua or Barbuda or Argentina or Armenia or Armenian or Aruba or Azerbaijan or Bahrain or Bangladesh or Barbados or Benin or Byelarus or Byelorussian or Belarus or Belorussian or Belorussia or Belize or Bhutan or Bolivia or Bosnia or Herzegovina or Hercegovina or Botswana or Brasil or Brazil or Bulgaria or Burkina Faso or Burkina Fasso or Upper Volta or Burundi or Urundi or Cambodia or Khmer Republic or Kampuchea or Cameroon or Cameroons or Cameron or Camerons or Cape Verde or Central African Republic or Chad or Chile or China or Colombia or Comoros or Comoro Islands or Comores or Mayotte or Congo or Zaire or Costa Rica or Cote d'Ivoire or Ivory Coast or Croatia or Cuba or Cyprus or Czechoslovakia or Czech Republic or Slovakia or Slovak Republic or Djibouti or French Somaliland or Dominica or Dominican Republic or East Timor or East Timur or Timor Leste or Ecuador or Egypt or United Arab Republic or El Salvador or Eritrea or Estonia or Ethiopia or Fiji or Gabon or Gabonese Republic or Gambia or Gaza or Georgia Republic or Georgian Republic or Ghana or Gold Coast or Greece or Grenada or Guatemala or Guinea or Guam or Guiana or Guyana or Haiti or Honduras or Hungary or India or Maldives or Indonesia or Iran or Iraq or Isle of Man or Jamaica or Jordan or Kazakhstan or Kazakh or Kenya or Kiribati or Korea or Kosovo or Kyrgyzstan or Kirghizia or Kyrgyz Republic or Kirghiz or Kirgizstan or Lao PDR or Laos or Latvia or Lebanon or Lesotho or Basutoland or Liberia or Libya or Lithuania or Macedonia or Madagascar or Malagasy Republic or Malaysia or Malaya or Malay or Sabah or Sarawak or Malawi or Nyasaland or Mali or Malta or Marshall Islands or Mauritania or Mauritius or Agalega Islands or Mexico or Micronesia or Middle East or Moldova or Moldovia or Moldovian or Mongolia or Montenegro or Morocco or Ifni or Mozambique or Myanmar or Myanma or Burma or Namibia or Nepal or Netherlands Antilles or New Caledonia or Nicaragua or Niger or Nigeria or Northern Mariana Islands or Oman or Muscat or Pakistan or Palau or Palestine or Panama or Paraguay or Peru or Philippines or Philipines or Phillipines or Phillippines or Poland or Portugal or Puerto Rico or Romania or Rumania or Roumania or Russia or Russian or Rwanda or Ruanda or Saint Kitts or St Kitts or Nevis or Saint Lucia or St Lucia or Saint Vincent or St Vincent or Grenadines or Samoa or Samoan Islands or Navigator Island or Navigator Islands or Sao Tome or Saudi Arabia or Senegal or Serbia or Montenegro or Seychelles or Sierra Leone or Slovenia or Sri Lanka or Ceylon or Solomon Islands or Somalia or South Africa or Sudan or Suriname or Surinam or Swaziland or Syria or Tajikistan or Tadzhikistan or Tadjikistan or Tadzhik or Tanzania or Thailand or Togo or Togolese Republic or Tonga or Trinidad or Tobago or Tunisia or Turkey or Turkmenistan or Turkmen or Uganda or Ukraine or Uruguay or USSR or Soviet Union or Union of Soviet Socialist Republics or Uzbekistan or Uzbek or Vanuatu or New Hebrides or Venezuela or Vietnam or Viet Nam or West Bank or Yemen or Yugoslavia or Zambia or Zimbabwe or Rhodesia).hw,ti,ab,cp. | 7413486 |
| 61 | ((developing or less* developed or under developed or underdeveloped or middle income or low* income or underserved or under served or deprived or poor*) adj (countr* or nation? or population? or world)).ti,ab. | 209736 |
| 62 | ((developing or less* developed or under developed or underdeveloped or middle income or low* income) adj (economy or economies)).ti,ab. | 1149 |
| 63 | (low* adj (gdp or gnp or gross domestic or gross national)).ti,ab. | 576 |
| 64 | (low adj3 middle adj3 countr*).ti,ab. | 29632 |
| 65 | (lmic or lmics or third world or lami countr*).ti,ab. | 14676 |
| 66 | transitional countr*.ti,ab. | 380 |
| 67 | or/58-66 | 7754184 |
| 68 | 57 and 67 **[CAPACITY BUILDING + HTA + LMIC]** | 3459 |
| 69 | capacity building/ | 6575 |
| 70 | exp medical education/ | 459668 |
| 71 | professional competence/ | 55107 |
| 72 | knowledge/ | 46839 |
| 73 | or/69-72 | 553059 |
| 74 | decision making/ | 303150 |
| 75 | health care policy/ | 186861 |
| 76 | medical research/ | 279490 |
| 77 | health services research/ | 68492 |
| 78 | or/74-77 | 810132 |
| 79 | 73 and 78 | 49761 |
| 80 | ((capacity or competenc* or knowledge or skill? or qualification? or education or training) adj6 (research evidence or scientific evidence or medical research or health research or health services research or health systems or health policy or health care policy or policy making or decision making)).ti,ab,kw. | 22746 |
| 81 | 79 or 80 | 70833 |
| 82 | ghana/ or ghana*.ti,ab,kw. | 24115 |
| 83 | 81 and 82 **[CAPACITY BUILDING + RESEARCH / POLICY + GHANA]** | 152 |
| 84 | 68 or 83 | 3597 |
| 85 | limit 84 to embase [Limit not valid in Ovid MEDLINE(R),Ovid MEDLINE(R) Daily Update,Ovid MEDLINE(R) In-Process,Ovid MEDLINE(R) Publisher; records were retained] | 2603 |
| 86 | limit 85 to yr="2005-Current" | 2431 |
| 87 | 86 use oemez | 1031 |
| 88 | 48 or 87 | 3991 |
| 89 | remove duplicates from 88 | 3373 |

**Other Source Searched**We searched for grey literature across search engines (i.e. Google Scholar, New York Academy of Medicine Grey Literature Report, and OpenGrey), international development agencies or foundations (e.g. Robert Wood Johnson Foundation, The Melinda and Bill Gates Foundation), and organizations/institutes involved in global health initiatives and capacity building strategies such as the Institute of Research for Poverty, Netherlands Development, etc. Lastly, we reviewed the reference lists of articles that met our inclusion criteria.
