# Supplementary material for: Developing a tool to assess the skills to perform a health technology assessment
Source: BMC Med Res Methodol. 2022 Mar 22;22:78. doi: 10.1186/s12874-022-01562-4 (PMC8939100; doi:10.1186/s12874-022-01562-4)
Supplement: Supplementary file 2 — Additional file 2. [file 12874_2022_1562_MOESM2_ESM.pdf]

Version 1. February 2021 - Instrument for the assessment of skills to conduct a Health Technology Assessment

This survey to assess the skills to conduct a Health Technology Assessment is freely available to be used for assessments or research. Please contact the authors if you would like to access a soft copy of the questions.

Recommended citation: Bidonde J, Meneses-Echavez JF, Peacocke E, *Instrument for the assessment of skills to conduct a Health Technology Assessment Version 1*. Oslo: Norwegian Institute of Public Health. February 2021.

The development of this survey was partially funded by the Norwegian Agency for Development Cooperation.

Welcome to this survey of skills to conduct a HTA. The findings will be presented in anonymous and aggregate form and will be used to inform the implementation of activities.

Please answer all questions. We ask you to answer them as honestly as possible and to the best of your ability.

Structure of the survey
This survey consists of the following four sections and must be completed in one sitting (you cannot save a survey that you have started). Please allocate 20-30 minutes to complete it.

General information
- Professional experience
- Experience with HTA

Core skills
- Clinical effectiveness
- Ethics
- Patient and Public Involvement
- Health economics

Soft skills
- Management
- Communication
- HTA governance

Future needs
- Skills you would like to develop in the future

Any questions, please contact **Elizabeth Peacocke (**efpe@fhi.no**), or WhatsApp +47 941 60 559**.

Thank you in advance for your time.

1) CONSENT

By completing the survey you agree for your answers to be analysed and that the results will be presented in anonymous form.

[instert text as agreed through ethical prcesses]

- ○ Yes
- ○ No

This box is shown in preview only.

The following conditions must be fulfilled for this question to be shown:

If the question “Do you consent to participate?” contains any of these alternatives
- “No”

These actions will take effect for the following alternatives:
- Take me out of the questionnaire : Display thank you note

- ○ Take me out of the questionnaire

GENERAL INFORMATION

3) * Full name

4) Please select your age range

- ○ Less than 30
- ○ 31-40
- ○ 41-50
- ○ 51-60
- ○ Older than 60 years

5) Gender

- ◯ Female
- ◯ Male
- ◯ Other
- ◯ Prefer not to say

6) Which language, other than English, can you work in? (e.g. to translate a paper, extract data, complete risk of bias)

7) What is your highest level of education?

- ◯ Bachelor
- ◯ Master
- ◯ Doctoral
- ◯ Other, please specify

8) Field of study (e.g. nursing)

9) What are your main area(s) of professional work experience? (e.g. public health)

10) How many years of professional experience do you have?

- ◯ <5
- ◯ 6-10
- ◯ 11-15
- ◯ 16-20
- ◯ More than 20

11) Name of your current employer

12) Which countries do you currently work with e.g. international collaborations?

13) * Have you ever been part of any governmental or ministerial committee?

- Yes
- No

14) If yes, what committee and how many years have/did you serve on it?

15) * Do you have experience in conducting research?

- Yes
- No

16) If yes, number of years

17) * Which of the following statements best describes your use of research• in your everyday work?

- I do not or very rarely use research
- I use research once in a while
- I am a research user/evidence based practitioner
- I work as a researcher

•Research: In this context we are referring to the consideration of study regarding a particular concern or problem using scientific methods.

18) * Have you ever undertaken a systematic review or other type of evidence synthesis (e.g. clinical guideline, mapping review)? If yes, how many?

- ○ No
- ○ Yes, 1-5
- ○ Yes, 6-10
- ○ Yes, more than 10

19) If yes, what type of review/guideline/analysis did you work on? (check all that apply)

- ☐ Systematic review of intervention
- ☐ Diagnostic review (DTA)
- ☐ Network Meta-Analysis (NMA)
- ☐ Scoping/mapping review
- ☐ Clinical guideline
- ☐ Other, please specify ____________

20) If yes, what best described your role(s)

- ☐ Project lead
- ☐ Senior responsible
- ☐ Stakeholder
- ☐ Project manager
- ☐ Advisor
- ☐ Researcher
- ☐ One of many collaborators

21) * Have you ever used the results of an HTA?

- ○ Yes
- ○ No

22) * Have you ever undertaken an economic evaluation? If yes, how many?

- ○ No
- ○ Yes, 1-5
- ○ Yes, 6-10
- ○ Yes, more than 10

23) If yes, what type of economic evaluation did you work on?

- ☐ Cost Effective Analysis (CEA)
- ☐ Cost Benefit Analysis (CBA)
- ☐ Cost Utility Analysis (CUA)
- ☐ Cost Minimisation Analysis (CMA)
- ☐ Cost Consequences Analysis (CCA)
- ☐ Other, please specity ________

24) If yes, what best described your role in the economic evaluation(s)?

- ☐ Project lead
- ☐ Senior responsible
- ☐ Stakeholder
- ☐ Project manager
- ☐ One of many collaborators
- ☐ Advisor

25) * Have you ever used the results of an economic evaluation?

- ○ Yes
- ○ No

You are now finishing the general information section. Once you click the "Next" button, you will not be able to go back to this section of the survey.

CORE SKILLS TO CONDUCT HTA

CLINICAL EFFECTIVENESS

For the following questions, answer as though you are *planning* an HTA.

28) * Indicate your level of comfort:

	No knowledge on this topic	I have heard of this topic but I don't feel confident to do it	Slightly confident to do it	I master this topic
Writing a structured research question according to the PICO acronym	○	○	○	○
Searching for the studies	○	○	○	○
Deciding when to conduct a new HTA or adapt an existing one	○	○	○	○
Developing a protocol and registration	○	○	○	○
Defining what data to extract	○	○	○	○
Creating data extraction forms	○	○	○	○
Conducting data extraction process (who will do it, how many reviewers)	○	○	○	○
Handling missing data	○	○	○	○
Extracting data that is presented graphically (e.g. pie charts, bar graphs in articles)	○	○	○	○

Answer as though you are *doing* an HTA.

29) * Searching for the studies, indicate your level of comfort with:

	No knowledge on this topic	I have heard of this topic but I don’t feel confident to do it	Slightly confident to do it	I master this topic
Electronic sources and databases to search for evidence	○	○	○	○
Grey literature: ongoing studies and unpublished data sources	○	○	○	○
Other search approaches (hand searching and citation snowballing)	○	○	○	○
How to structure a search strategy	○	○	○	○
Controlled vocabulary and text words (MeSH terms, Boolean operators) i.e. how to structure a search strategy, combining terms and concept blocks	○	○	○	○
Search filters and limits (languages, range of years, type of studies, setting/geographical location)	○	○	○	○
Reference management and data management software (e.g. EndNote, DistillerSR, Rayyan, or other)	○	○	○	○

30) * Collecting data, indicate your level of comfort:

	No knowledge on this topic	I have heard of this topic but I don’t feel confident to do it	Slightly confident to do it	I master this topic
Defining what data to extract	○	○	○	○
Creating data extraction forms	○	○	○	○
Completing the data extraction process (i.e. who will do it, how many reviewers)	○	○	○	○
Handling missing data	○	○	○	○
Extracting data that is presented graphically (e.g. pie charts, bar graphs in articles)	○	○	○	○
Following data management requirements (e.g. individual studies, national registries)	○	○	○	○

31) * Critical appraisal skills, indicate your level of comfort:

	No knowledge on this topic	I have heard of this topic but I don’t feel confident to do it	Slightly confident to do it	I master this topic
Identifying validated tools to critically appraise scientific literature	○	○	○	○
Recognizing the most important domains to appraise in the different study designs	○	○	○	○
Understanding of the procedure through which critical appraisal should be undertaken	○	○	○	○

32) * Have you ever critically appraised the quality of any of the following? (choose all that apply)

	Yes	No	If yes, please add name of the instrument(s) e.g. Cochrane RoB, CHEERS, ROBINS-I
Randomized controlled trial	○	○	
Non-randomized controlled trial	○	○	
Observational study	○	○	
Diagnostic/Prognostic study	○	○	
Qualitative study	○	○	
Systematic review of interventions	○	○	
Economic evaluations	○	○	
Clinical practice guideline	○	○	

33) * Summarizing study characteristics and preparing for synthesis, indicate your level of comfort:

	No knowledge on this topic	I have heard of this topic but I don’t feel confident to do it	Slightly confident to do it	I master this topic
Summarizing the characteristics of each study	○	○	○	○
Determining comparability across studies (i.e. clinical, statistical, and methodological heterogeneity)	○	○	○	○
Identifying relevant comparisons from the included studies	○	○	○	○

34) * Data synthesis and analysis, indicate your level of comfort with:

	No knowledge on this topic	I have heard of this topic but I don't feel confident to do it	Slightly confident to do it	I master this topic
Data analysis software (RevMan, R, Stata, Excel)	○	○	○	○
Effect measures for dichotomous and continuous data (risk ratio, mean difference, etc.)	○	○	○	○
Meta-analysis	○	○	○	○
Meta-regression	○	○	○	○
Addressing heterogeneity	○	○	○	○
Subgroup and sensitivity analysis	○	○	○	○
Narrative synthesis when a meta-analysis is not possible (e.g. logical categories)	○	○	○	○
Network meta-analysis	○	○	○	○
Principles of a network meta-analysis (transitivity or evidence network)	○	○	○	○
Geometry of the treatment network	○	○	○	○

35) * Qualitative evidence synthesis (QES), indicate your level of comfort in the:

	No knowledge on this topic	I have heard of this topic but I don't feel confident to do it	Slightly confident to do it	I master this topic
Use of QES in HTA	○	○	○	○
Methods for QES: thematic synthesis, framework synthesis, and meta-ethnography	○	○	○	○
Transformation of large amounts of data	○	○	○	○
Writing skills	○	○	○	○
Balanced description and interpretation	○	○	○	○
Reflexivity (e.g. examining your own position in a situation)	○	○	○	○

36) * Grading the certainty of the evidence, indicate your level of comfort with:

	No knowledge on this topic	I have heard of this topic but I don't feel confident to do it	Slightly confident to do it	I master this topic
Summary of findings (SoF) tables or evidence profiles	○	○	○	○
Ranking the outcomes for SoF tables or evidence profiles	○	○	○	○
Generating SoF tables or evidence profiles	○	○	○	○
Various approaches for assessing the certainty of a body of evidence	○	○	○	○
GRADE-Pro GDT software	○	○	○	○

ETHICS

37) * Ethics, indicate your level of comfort with:

	No knowledge on this topic	I have heard of this topic but I don't feel confident to do it	Slightly confident to do it	I master this topic
Ethical issues concerning technologies	○	○	○	○
Models of moral philosophy (utilitarianism, casuistic, personalism) and theories of justice	○	○	○	○
Bioethical issues and concepts (self-determination, privacy, inform consent, etc.)	○	○	○	○

PUBLIC AND PATIENT INVOLVEMENT (PPI)

38) * Public and Patient Involvement (PPI) (adapted from PPEET Tool), indicate your level of comfort with:

	No knowledge on this topic	I have heard of this topic but I don't feel confident to do it	Slightly confident to do it	I master this topic
Public and patient engagement (PPI) in HTA	○	○	○	○
Mode of Engagement•	○	○	○	○
Identifying and recruiting those most affected by the decision	○	○	○	○
Use of input from PPI	○	○	○	○

•Mode of engagement:

1. Communication = Public & Patient (PP) receive information but have no role in contributing to HTA;
2. Consultation = PP provide their views, thoughts, opinions but there is no commitment to act on them;
3. Collaboration = PPI are engaged to influence the production of the HTA (e.g. commenting, advising, voting;
4. Co-production = PP are equal members of the HTA development team and participate in all steps of the HTA development process

HEALTH ECONOMICS

39) * Health Economics (adapted from IDSI's economic tool), indicate your level of comfort with:

	No knowledge on this topic	I have heard of this topic but I don't feel confident to do it	Slightly confident to do it	I master this topic
Economic evaluation of health interventions, for example using cost-effectiveness analysis, cost benefit analysis	○	○	○	○
Measuring the costs of health interventions, for example through micro-costing, activity-based costing	○	○	○	○
Measuring the economic burden of disease, for example through cost-of-illness studies, macroeconomic modelling	○	○	○	○
Measuring health equity, for example estimating the incidence of catastrophic health expenditure, benefit-incidence analysis, extended cost-effectiveness analysis	○	○	○	○
Measuring the efficiency of health systems or health service providers, for example using data envelopment analysis, stochastic frontier analysis	○	○	○	○
Measuring the preferences of health workers or patients, for example using discrete choice experiments, contingent valuation	○	○	○	○
Measuring health utilities and health-related quality of life, for example collecting and analysing individual-level data using EQ-5D or SF-6D instruments	○	○	○	○
Formal policy analysis, for example using the framework "Problem identification-Evidence gathering-Solution analysis-Policy recommendation"	○	○	○	○
Political economy analysis – national, sector-based, problem-based	○	○	○	○
Decision analytic modelling, for example using decision trees, Markov models, discrete event simulation	○	○	○	○
Budget impact analysis	○	○	○	○

You are now finishing the "Core skills" section. Once you click the "Next" button, you will not be able to go back to this section of the survey.

SOFT SKILLS TO CONDUCT HTA

42) * Rate your skills from none (1) to high (4)

	1 none	2 low	3 moderate	4 high
Project coordination and management	○	○	○	○
Planning and leading project meetings/activities	○	○	○	○
Prepare a master flowchart with the sequencing of activities and milestones	○	○	○	○
Resourcefulness (knowing options, finding solutions)	○	○	○	○
Dialogue and negotiation skills	○	○	○	○
Networking	○	○	○	○
Communication of concerns, disagreements, changes, successes, etc.	○	○	○	○
Dealing with conflict and solving problems when they arise	○	○	○	○
Flexibility and adaptation (i.e. dealing with interferences or back orders)	○	○	○	○
Leadership	○	○	○	○
Mentorship and collaboration	○	○	○	○
Working in a multidisciplinary team	○	○	○	○
Acknowledge good work	○	○	○	○

You are now finishing the "Soft skills" section. Once you click the "Next" button, you will not be able to go back to this section of the survey.

FUTURE NEEDS

In this section we are interested to know what core or soft skills you would like to develop further.

SEARCHING FOR STUDIES AND SYNTHESIS OF CLINICAL EVIDENCE

Which of the following skills do you consider most important for you to develop in the future? Place most important first.

45) Please order the following skills in order of importance for you to develop

	1	2	3	4	5	6	7	8
Searching for the studies	○	○	○	○	○	○	○	○
Collecting data	○	○	○	○	○	○	○	○
Study appraising skills	○	○	○	○	○	○	○	○
Summarizing study characteristics and preparing for synthesis	○	○	○	○	○	○	○	○
Data synthesis and analysis	○	○	○	○	○	○	○	○
Network meta-analysis	○	○	○	○	○	○	○	○
Qualitative evidence synthesis (QES)	○	○	○	○	○	○	○	○
Grading the certainty of the evidence	○	○	○	○	○	○	○	○

HEALTH ECONOMICS

Which of the following skills do you consider most important for you to develop in the future? Place most important first.

46) Please order the following skills in order of importance for you to develop

	1	2	3	4	5	6	7	8	9	10	11
Economic evaluation of health interventions (cost-effectiveness analysis, cost benefit analysis)	○	○	○	○	○	○	○	○	○	○	○
Measuring the costs of health interventions (micro costing, activity-based costing)	○	○	○	○	○	○	○	○	○	○	○
Measuring the economic burden of disease (cost-of-illness studies, macroeconomic modelling)	○	○	○	○	○	○	○	○	○	○	○
Measuring health equity (estimating the incidence of catastrophic health expenditure, benefit-incidence analysis, extended cost-effectiveness analysis)	○	○	○	○	○	○	○	○	○	○	○
Measuring the efficiency of health systems or health service providers (using data envelopment analysis, stochastic frontier analysis)	○	○	○	○	○	○	○	○	○	○	○
Measuring the preferences of health workers or patients (discrete choice experiments, contingent valuation)	○	○	○	○	○	○	○	○	○	○	○
Measuring health utilities and health-related quality of life (collecting and analyzing individual-level data using EQ-5D or SF-6D instruments)	○	○	○	○	○	○	○	○	○	○	○
Formal policy analysis, for example using the framework "Problem identification-Evidence gathering-Solution analysis-Policy recommendation"	○	○	○	○	○	○	○	○	○	○	○
Political economy analysis – national, sector-based, problem-based	○	○	○	○	○	○	○	○	○	○	○
Decision analytic modelling (decision trees, Markov models, discrete event simulation)	○	○	○	○	○	○	○	○	○	○	○
Budget impact analysis	○	○	○	○	○	○	○	○	○	○	○

ETHICS

Which of the following ethics' areas do you consider most important for you to develop in the future?

47) Please order the following skills in order of importance for you to develop

	1	2	3
General ethical issues concerning technologies	○	○	○
Models of moral philosophy (utilitarianism, casuistic, personalism) and theories of justice	○	○	○
Bioethical issues and concepts (self-determination, privacy, inform consent, etc.)	○	○	○

PATIENT AND PUBLIC INVOLVEMENT (PPI)

Which of the following PPI areas do you consider most important for you to develop in the future?

48) Please order the following in order of importance

	1	2	3
General knowledge of PPI in HTA	○	○	○
Identifying and recruiting those most affected by the decision	○	○	○
Using of input from PPI	○	○	○

49) Are there any future needs that we have not covered above? Please specify:

0/4000

© Copyright www.questback.com. All Rights Reserved.
Trial Essentials for free - Click here to create your survey today.
