# Supplementary material for: Developing a tool to assess the skills to perform a health technology assessment
Source: BMC Med Res Methodol. 2022 Mar 22;22:78. doi: 10.1186/s12874-022-01562-4 (PMC8939100; doi:10.1186/s12874-022-01562-4)
Supplement: Supplementary file 3 — Additional file 3. [file 12874_2022_1562_MOESM3_ESM.docx]

**Additional File 2.** Flow diagram PRISMA 2020 (17)

**Identification of studies via other methods**

**Identification of studies via databases**

Records removed *before screening*:

Duplicate records removed (n = 870)

Records marked as ineligible by automation tools (n = 0)

Records removed for other reasons (n = 325)

Records identified from:

Websites and organizations (n = 22)

Citation searching (n = 10)

etc.

Records identified from:

Databases (n = 3871)

MEDLINE (2960)

Embase (1031)

Web of Science (263)

ERIC (155)

**Identification**

Records excluded

(n = 2651)

Records screened

(n = 2676)

**Screening**

Reports sought for retrieval

(n = 25)

Reports not retrieved

(n = 0)

Reports sought for retrieval

(n = 32)

Reports not retrieved

(n = 0)

Reports excluded:

No focus on HTA (n = 1)

No tool provided (n = 16)

Setting (n = 2)

Reports assessed for eligibility

(n = 32)

Reports excluded:

No focus on HTA (n = 17)

No tool provided (n = 6)

Reports assessed for eligibility

(n = 25)

**Included**

Reports included in review

(n = 15)
