# Supplementary material for: Developing a tool to assess the skills to perform a health technology assessment
Source: BMC Med Res Methodol. 2022 Mar 22;22:78. doi: 10.1186/s12874-022-01562-4 (PMC8939100; doi:10.1186/s12874-022-01562-4)
Supplement: Supplementary file 4 — Additional file 4. [file 12874_2022_1562_MOESM4_ESM.docx]

**Additional File 3 – Tools identified by the mapping review**

| **Author/ (setting)**  **Title** | **Tool Category** | | **Goal(s)** | **Study design / evaluation type** | **Target group / evaluation level** |
| --- | --- | --- | --- | --- | --- |
|  | **HTA/CB** | **EBP** |  |  |  |
| Africa Evidence Network 2017 (32) / (Africa)  Capacity in Africa: The results of a survey on support for and production of evidence maps and evidence syntheses, including systematic reviews |  | x | a. to document the evidence synthesis capacity within Africa,  b.to explore the existing needs and support needed | Cross-sectional /capacity assessment | Researchers and stakeholders |
| Bates et al. 2006 (27) / (Ghana)  Evaluating Health Research Capacity Building: An Evidence-Based Tool |  | x | a. to describe the process of developing an evidence-based tool | Literature review and tool development/capacity assessment | Policy makers and researchers/organizational |
| Doherty et al. 2016 (33) / (LMICs)  Effective capacity building strategies for HTA: a rapid review of international experience | x |  | a. to review the evidence on capacity building activities for HTA in South Africa | Rapid review/process evaluation | Researchers and stakeholders /organizational |
| Franzen et al. 2017 (28) / (LMICs)  Health research capacity development in low- and middle-income countries: reality or rhetoric? A systematic meta-narrative review of the qualitative literature |  | x | a.to identify and examine the main approaches and trends in health research capacity development  b. to consolidate key thinking to identify a more coherent approach | Systematic review/NA | Unclear/NA |
| Gadsby et al. 2011 (24) / (LMICs)  Research capacity strengthening donor approaches to improving and assessing its impact in low- and middle-income countries | x | x | a. to inform the understanding of how research capacity is being strengthened, and to how this might consider the effectiveness of these initiatives. | Literature review and interviews/NA | Representatives from organizations involved in research capacity strengthening/organizational |
| Huber et al. 2015 (25) / (HIC and LMICs)  Tools and instruments for needs assessment, monitoring and evaluation of health research capacity development activities at the individual and organizational level: a systematic review | x | x | a. to summarize evidence on tools and instruments for the needs assessment, monitoring and evaluation (NaME) of health research capacity development (HRCD) activities at the individual, team and the organizational levels | Systematic review/NA | Researchers and stakeholders/NA |
| Luz et al. 2018 (34) / (LMICs)  Identifying priority technical and context-specific issues in improving the conduct, reporting and use of health economic evaluation in LMICs | x |  | a. to identify the priority issues that impede the conduct, reporting and use of economic evaluation in LMICs b.to explore potential solutions for these priority issues | Cross-sectional study/process evaluation | Researchers and stakeholders/organizational |
| Minja et al. 2011 (26) / (LMICs)  Impact of Health Research Capacity Strengthening in Low- and Middle-Income Countries: The Case of WHO/TDR Programmes | x | x | a. to assess the impact of the UNICEF/UNDP/World Bank /WHO Special Programme for Research and Training in Tropical Diseases on the development of tropical disease research and research capacity in disease endemic countries | Cross-sectional study (mixed methods)/program evaluation | Grant recipients (researchers)/ individual and organizational |
| Oliver et al. 2015 (29) / (LMICs)  Capacity for conducting systematic reviews in low- and middle-income countries: a rapid appraisal |  | x | a.to evaluate the capacity for conducting systematic reviews in low- and middle-income countries | Cross-sectional study/capacity assessment | Researchers and stakeholders/organizational |
| Ramos et al. 2003 (30) / (Global)  Fresno Test |  | x | a. to assess the level at which EBM skills are utilized in practice | Research tool/outcome evaluation | Practitioners and researchers/individual |
| Tantivess et al. 2017 (35) / (LMICs)  HTA capacity development in LMICs: Experiences from the international units of HITAP and NICE | x |  | a. to present lessons from the Health Intervention and Technology Assessment Program in Thailand, and the UK, from their capacity building programs in India, Colombia, Myanmar, the Philippines, and Vietnam | Literature review/capacity assessment | Policy makers and researchers/organizational |
| Tilson et al. 2011 (31) / (Global)  Sicily statement |  | x | a. to provide guidance for purposeful classification and development of tools to assess EBP learning | Expert statement/report(?)/outcome evaluation | Practitioners, researchers, and educators |
| United Nations Development Programme 2008(36) / (Global)  Capacity Assessment Methodology: Users’ Guide | x |  | a. to present a step-by-step guide to conducting a capacity assessment using the UNDP Capacity Assessment Methodology | Organizational report/capacity assessment | Those conducting capacity assessments/NA |
| Watson-Grant et al. 2018 (37) / (LMICs)  Framework and Toolkit to Strengthen Evaluation Capacity | x |  | a. to review literature on the Evaluation Capacity Strengthening framework, and to provide a guidance document for assessing and planning evaluation capacity strengthening | Organizational report/capacity assessment | Program evaluators, researchers, and stakeholders/organizational |
| WHO 2015 (38) / (Global)  2015 Global Survey on HTA by National Authorities | x |  | a. to present main findings from The WHO 2015 Global Survey on HTA | Cross-sectional study/capacity assessment | Practitioners, researchers, policymakers, and decision-makers |

Key: CB capacity building; EBM: evidence-based medicine: EBP: evidence-based practice; HITAP: Health Intervention and Technology Assessment Program; HTA: health technology assessment; LMICs: low and middle income country; NA: no available; NICE: National Institute for Health and Care Excellence; UNDP: United Nations Development Program; UNICEF: United Nations Children’s Fund; WHO: World Health Organization.
